# Crack Identification in Necked Double Shear Lugs by Means of the Electro-Mechanical Impedance Method

**DOI:** 10.3390/s21010044

**Published:** 2020-12-23

**Authors:** Markus Winklberger, Christoph Kralovec, Christoph Humer, Peter Heftberger, Martin Schagerl

**Affiliations:** 1 Institute of Structural Lightweight Design, Johannes Kepler University Linz, Altenberger Str. 69, 4040 Linz, Austria; christoph.kralovec@jku.at (C.K.); christoph.humer@jku.at (C.H.); martin.schagerl@jku.at (M.S.); 2 Ro-Ra Aviation Systems GmbH, Gewerbepark 8, 4861 Schörfling am Attersee, Austria; p.heftberger@ro-ra.com; 3 Christian Doppler Laboratory for Structural Strength Control of Lightweight Constructions, Johannes Kepler University Linz, Altenberger Str. 69, 4040 Linz, Austria

**Keywords:** necked double shear lug, aircraft structure, fatigue, model-based, crack identification, electro-mechanical impedance method, coupled-field FEM

## Abstract

This contribution investigates fatigue crack detection, localization and quantification in idealized necked double shear lugs using piezoelectric transducers attached to the lug shaft and analyzed by the electro-mechanical impedance (EMI) method. The considered idealized necked lug sample has a simplified geometry and does not includes the typical bearing. Numerical simulations with coupled-field finite element (FE) models are used to study the frequency response behavior of necked lugs. These numerical analyses include both pristine and cracked lug models. Through-cracks are located at 90${}^{\circ }$ and 145${}^{\circ }$ to the lug axis, which are critical spots for damage initiation. The results of FE simulations with a crack location at 90${}^{\circ }$ are validated with experiments using an impedance analyzer and a scanning laser Doppler vibrometer. For both experiments, the lug specimen is excited and measured using a piezoelectric active wafer sensor in a frequency range of 1 kHz to 100 kHz. The dynamic response of both numerical calculations and experimental measurements show good agreement. To identify (i.e., detect, locate, and quantify) cracks in necked lugs a two-step analysis is performed. In the first step, a crack is detected data-based by calculating damage metrics between pristine and damaged state frequency spectra and comparing the resulting values to a pre-defined threshold. In the second step the location and size of the detected crack is identified by evaluation of specific resonance frequency shifts of the necked lug. Both the search for frequencies sensitive to through-cracks that allow a distinction between the two critical locations and the evaluation of the crack size are model-based. This two-step analysis based on the EMI method is demonstrated experimentally at the considered idealized necked lug, and thus, represents a promising way to reliably detect, locate and quantify fatigue cracks at critical locations of real necked double shear lugs.

## 1. Introduction

Lug type joints represent a common way to connect structural parts in many fields of engineering, e.g., aircraft and automotive. Compared to riveted and adhesively bonded joints, lug joints are detachable connections which allow an easy assembling and disassembling. Furthermore, without clamping of the fork these connections act as pivot points and avoid the introduction of bending moments in the surrounding structure. However, one major disadvantage of such structural parts is their poor fatigue strength, as high stress concentrations and fretting at the bolt hole of lugs can lead to early crack initiation [1]. For the specific shape of necked double shear lugs the situation is even more critical. Crack initiation does not only occur at the bolt hole but also on the outer surface at the transition region between shaft and largest diameter of the necked lug [2]. If such lugs are used at single load path locations, their complete failure can lead to catastrophic events. Given the obvious advantages and disadvantages of lug joints and their essential role for the safe operation of vehicles a reliable method to detect damages in such components is of paramount interest.

Structural Health Monitoring (SHM) promises to solve such safety issues of necked double shear lugs by retaining the simple geometry and advantageous assembling possibilities. SHM developed from non-destructive testing (NDT) and additionally enables the onboard monitoring of mechanical structures during operation. This is possible due to integrated and lightweight sensors mounted directly and permanently on the monitored structure. Sensor readings are processed and analyzed according to the applied SHM method [3]. Examples of state-of-the-art monitoring methods are guided waves [4,5,6,7], conductive surface layers [8,9,10], direct measurements of the electrical impedance of a structure [11] and the electro-mechanical impedance (EMI) method [12,13,14,15].

Piezoelectric transducers are used in a wide range of SHM application due to their small size, low cost, the possibility to be embedded into the structure and the fact that they can act as sensor and actuator simultaneously. SHM methods based on piezoelectric transducers can be classified in passive, such as acoustic emission [16], and active methods, such as guided waves [17,18,19] and the EMI method [20]. The acoustic emission proved to be successful for, e.g., the monitoring of rolling contact fatigue damage [21], fatigue cracks in thin metallic plates [22] and additively manufactured components [23]. However, the interpretation of acoustic emission parameters for damage evaluation in complex geometries exposed to operational loads still remains as a challenge to be solved.

SHM based on guided waves can be used to monitor large thin-walled structures, as guided waves have the ability to travel over long distances with little loss of energy [24] and strongly interact with structural changes. Hence, guided waves can be used to detect structural damages of many kinds, such as cracks in riveted plates [25], delaminations in composite materials [26], debondings in carbon fiber reinforced concrete structures [27], among many others. However, guided wave-based SHM methods need advanced signal processing to correctly interpret the complex signals back-scattered from structural boundaries and potential damages, which is particularly challenging for complex and small structural components as the necked double shear lug considered in the present investigation.

Among the many SHM methods introduced in recent years, some have been applied to detect and track cracks in lugs and similar structures. A guided wave-based method in combination with a particle filter was used by Yuan et al. [28] for online monitoring of crack propagation in straight attachment lugs. The lug joint was equipped with multiple piezoelectric sensors on both sides and signals were measured in the pitch-catch approach. This proposed method facilitates multiple piezoelectric transducers in a certain distance and thus, needs relatively large space, i.e., for small components where multiple sensors cannot be applied this method is hardly applicable. Another SHM approach using piezoelectric sensors to track the crack growth process was applied by Lim and Soh [29] on simple aluminum beams under cyclic loading. There they used the electro-mechanical impedance method to estimate the remaining fatigue life by facilitating just a single piezoelectric patch bonded to the beams surface. In a recently published article Wang et al. [30] used a similar specimen and test setup. However, in both articles [29,30] it is pointed out that the proof-of-concept study of the beam has yet to be expanded to real-life structural components.

This contribution investigates the crack detection capabilities on comparatively small and simplified necked lugs with the EMI method, as a first step towards the damage evaluation of real lugs at operational conditions. By employing the EMI method, only one piezoelectric wafer active sensor (PWAS) is sufficient to excite different resonances of the structure and to measure its response at the same time. Hence, little space, cabling and additional weight is required on the structure.

Related to the application of a single PWAS this contribution aims to answer the following questions, c.f. [7] (p. 809ff): (i) Is it possible to detect cracks in necked lugs with the EMI method at all? (ii) Which concept should be used to identify the crack? Are damage indicators as damage metrics (e.g., RMSD, MAPD, CCD) or spectral features (e.g., specific resonant peak amplitudes, resonant frequencies, mode shapes) sensitive enough to reliably detect the cracks? (iii) Which frequency range is most sensitive to the crack and gives most reliable results. (iv) Is it possible to distinguish between different locations of crack initiation and crack sizes in necked lugs with just one PWAS?

**Figure 1:**
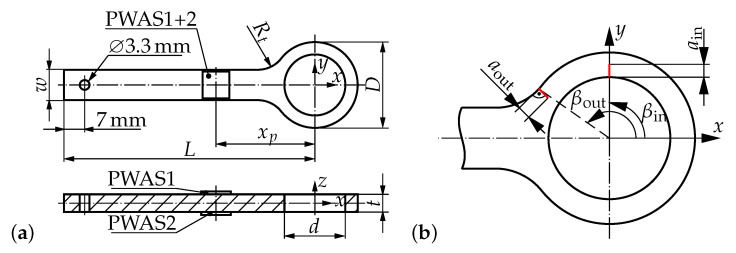
Geometry of simple lug, (**a**) dimensions of the lug and PWAS positions; (**b**) size and locations of investigated through-cracks.

The present investigation tries to answer these questions for a simplified necked lug geometry, which is similar in shape and dimensions to lugs in real application. For such lugs exposed to cyclic loading, crack initiation was observed in a previous study [2] to take place either at the inside at the bolt hole (90${}^{\circ }$ to the loading direction) or at the outside at the transition region between bolt hole and shaft, see Figure 1b. Typical crack types observed in fatigue loaded lugs are through-cracks, quarter circular and elliptical corner cracks. However, in this study only through-cracks are investigated as they represent a worst case scenario for cracks in lugs [1]. Consequently, these critical locations of crack initiation and crack shape are investigated in the present work. Coupled-field finite element (FE) models are developed to simulate the PWAS response for each crack location. Additionally, the results obtained for cracks at the bolt hole are validated and compared with measurements of an impedance analyzer (IMA) and a scanning laser Doppler vibrometer (SLDV). Finally, conclusions are drawn about the crack monitoring capabilities with EMI measurements of a single PWAS on a necked lug by combining all these results.

## 2. Lug Geometry and Material

The investigated idealized lug has a simple flat geometry, see Figure 1. The dimensions are given as D=29mm, d=20.64mm, L=85mm, w=10.31mm, t=6mm and $R_{t}=10\mathrm{mm}$, which are similar to necked double shear lugs in real applications, e.g., lugs of interior tie-rods in aircraft [2].

The lug specimen is milled out of aluminum EN-AW 7075 plate material. The hole with a diameter of 3.3 mm in the lugs shaft was necessary for clamping of the lug during milling. In contrast to the flat lug considered in this contribution lugs in real applications are commonly made by turning with a following milling process. Furthermore, necked double shear lugs in real applications usually have a threaded shaft and are equipped with spherical bearings [31]. However, the simplified shape of the chosen lug has multiple advantages for presented investigations: (i) the production effort is minimized, (ii) the simple shape can be easily meshed for FE simulations, (iii) the application of PWAS is eased due to flat surfaces and (iv) the possibility to use simple analytical approaches for investigation. Additionally, no spherical bearing is put into the lug hole of the investigated necked double shear lug to avoid uncertainty factors such as unknown additional damping and residual stresses. Such idealized lug was chosen to isolate the effects of growing cracks, and hence, to be able to develop the basic principles of a methodology for crack identification in these components. Thus, the present study represents a first step to identify crack monitoring features at an idealized setup before adding further complexity until crack identification in operated necked double shear lugs is achieved.

Figure 1b depicts the investigated critical locations of crack initiation, which are chosen based on a recently published profound analysis of the lugs fatigue behavior [2]. The specified angles $\beta_{\mathrm{in}}=90{}^{\circ }$ and $\beta_{\mathrm{out}}=145{}^{\circ }$ define the locations of crack initiation at the inside and outside surface of the lug with respect to the *x*-axis. Independent of the location the investigated through-cracks in this study are defined normal to the corresponding surface of crack initiation. Individual investigations of each crack location (only one crack in the lug at a time) are performed in FE simulations. For experimental measurements a crack at a location of $\beta_{\mathrm{in}}=90{}^{\circ }$ and a length of 2 mm is introduced using a mechanical fret saw. The lengths of the through-cracks were defined to be rather large in order to obtain significant results to prove the concept of crack detection and identification in necked double shear lugs. Therefore, in FE simulations the crack lengths $a_{\mathrm{in}}=a_{\mathrm{out}}$ = 1 mm–3 mm are chosen for the present investigations. However, for a fatigue cyclic loading these cracks are expected to be still within the stable crack growth regime [1,2].

Two PWAS were applied on the top and bottom side of the lug’s shaft at $x_{p}=33.5\mathrm{mm}$ away from the bolt axis, see Figure 1. The two component epoxy adhesive Loctite EA 9466 was used to apply both PWAS. However, only PWAS1 is used (exited and measured in order to detect the defined cracks with the EMI method) in the present study.

## 3. Finite Element Simulations

FE simulations are performed in Abaqus 2019 to numerically calculate the resonance mode shapes as well as the impedance spectra captured by the PWAS. Furthermore, velocity results of parts of the lug are used to get mean transfer functions comparable with results of the SLDV.

### 3.1. Definition of FE Models and Analysis Setup

**Figure 2:**
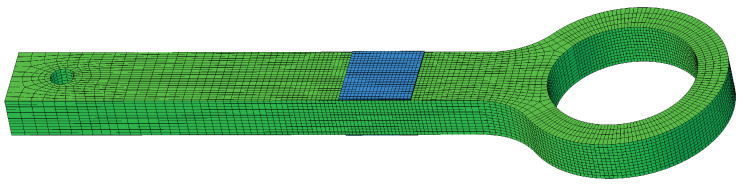
Mesh of complete assembly of pristine necked lug FE model.

A three dimensional FE model of the simple necked lug with a PWAS mounted to the upper and lower surface of the shaft is set up, see Figure 2. All dimensions including the position of the two PWAS are given in Figure 1a. To simulate the through-cracks, open seams are defined at two locations, see Figure 1b. The mesh of the lug exclusively consists of 8-node linear brick elements. For the PWAS special 8-node linear piezoelectric (coupled-field) brick elements are used. The overall mesh consists of 30,852 elements with an average edge length of 0.6 mm, see Figure 2.

The models with an inside crack at $\beta_{\mathrm{in}}=90{}^{\circ }$ and an outside crack at $\beta_{\mathrm{out}}=145{}^{\circ }$ incorporate an identical mesh as the pristine FE model except the additional nodes at the crack locations, due to the defined seams.

For the lug a linear elastic material model is defined according to the material data sheet with a Young’s modulus and a Poisson’s ratio of E=71.7GPa and ν=0.33, respectively. The density for aluminum is set to $\rho =2810\mathrm{kg}/m^{3}$. The used Rayleigh damping coefficients for aluminum are $\alpha =293.215\;s^{-1}$ and $\beta =4.12624\times 10^{-10}\;s$, which are empirical values found by manual fitting of experimental results [32]. The piezoelectric material behavior of the PWAS polarized in *z*-direction is implemented in Abaqus 2019 by providing three matrices: the piezoelectric matrix $\left[d\right]$, the orthotropic elasticity matrix $\left[c^{E}\right]$ at constant electric field and the electric permittivity matrix $\left[\varepsilon^{S}\right]$ at constant strain. The assumptions used to form the piezoelectric matrix $\left[d\right]$ and the orthotropic elasticity matrix are taken from [33] (p. 44f). The electric permittivity matrix at constant strain is defined according to [7] (p. 27ff). The PWAS’ material properties are taken from the supplier’s datasheet and are given in Table 1.

The PWAS are connected to the lug upper and lower surface using tie constraints. On the lower surface of each PWAS a constant electric potential of 0 V is applied. On the upper surface of just one of the two PWAS a harmonic electric potential with an amplitude of 5 V is defined (value in the same order of magnitude as experimental excitation; due to the investigation of normalized results the deviation of the excitation is negligible for little damped structures), which results in a combined axial and bending (around the *y*-axis) excitation of the shaft.

The cracks of length 2 mm at locations $\beta_{\mathrm{in}}=90{}^{\circ }$ and $\beta_{\mathrm{out}}=145{}^{\circ }$ are defined using the Abaqus seam functionality. Nodes at defined seams are duplicated, which creates an idealized crack without any gap [35]. Contact forces between crack faces are neglected.

The Abaqus simulation includes a coupled-field FE model within a direct-solution steady-state dynamics analysis (for further information see [35]). With this type of simulation the steady-state response of a harmonic loading is calculated for a given set of frequencies. The chosen frequency range is 1 kHz to 100 kHz. Additionally, in order to compare the simulations with the experimental measurements the same step size of 125 Hz was chosen. Hence, for each model 793 uniformly distributed frequency responses were calculated.

### 3.2. Processing of Raw FE Field Output

The raw FE field output cannot be directly used for comparison with experimental results [7]. Therefore, to compare the electrical impedance responses in this paper the conductance

(1) G(ω)=ReY(ω)=ReI(ω)U(ω),

is calculated using the exciting voltage U(ω) and the resulting current I(ω) for each specified angular frequency ω=2πf. As already mentioned above the defined boundary condition at the upper surface of PWAS1 is a harmonic electric potential U(ω) with an amplitude of $\hat{U}=5V$. The complex current I(ω) results from the derivation with respect to time of the harmonic electric charge *Q*. Thus, in the frequency domain it can be calculated with $I\left(\omega \right)=j\omega \sum_{n=1}^{N_{\mathrm{IMA}}}Q_{n}$, where $j=\sqrt{-1}$ is the imaginary unit and $N_{\mathrm{IMA}}$ is the number of nodes on the upper surface of PWAS1 [7]. For each node *n* of the upper surface of PWAS1 in the FE model the electrical charge $Q_{n}$ is provided as field output (reactive electrical nodal charge RCHG) by Abaqus [35]. However, Abaqus uses the constitutive equations without considering electric losses [35], see first two terms of Equation (2). To calculate more accurate results the electrical damping has to be considered, which is given by the dielectric loss factor δ [13]. According to [7] the piezoelectric constitutive equation with considering electrical damping for the electrical displacement $D_{i}$ is calculated for each material direction i,k,l=1,2,3 with

(2) $$D_{i}=\underset{\begin{array}{c} \mathrm{used}\;\mathrm{by}\;\mathrm{Abaqus}\\ \mathrm{to}\;\mathrm{calculate}\;Q_{n} \end{array}}{\underset{︸}{d_{ikl}T_{kl}+\varepsilon_{ik}^{T}E_{k}}}-\underset{\begin{array}{c} \mathrm{electrical}\\ \mathrm{damping} \end{array}}{\underset{︸}{j\delta \varepsilon_{ik}^{T}E_{k}}},$$

where $d_{ikl}$ is the piezoelectric matrix, $T_{kl}$ is the stress tensor and $\varepsilon_{ik}^{T}$ is the permittivity matrix at constant stress. Assuming a constant electrical field between the PWAS surface electrodes (with area $A_{p}$) that is only oriented in *z*-direction ($E_{3}=\hat{U}/t_{p}$) and normal to $A_{p}$ the electrical charge is calculated with [36]

(3) $$Q=\underset{A_{p}}{\;\int \int \;}D_{3}\;dA.$$

Hence, by inserting Equation (2) into Equation (3) and replacing the double integral by the sum of the numerically calculated $Q_{n}$ yields the current I(ω) corrected by the electrical damping

(4) $$I\left(\omega \right)=j\omega \left[\sum_{n=1}^{N_{\mathrm{IMA}}}\left(Q_{n}\right)-j\delta \varepsilon_{33}^{T}\frac{\hat{U}}{t_{p}}A_{p}\right].$$

Dielectric loss factor δ, permittivity $\varepsilon_{33}^{T}$, and the thickness of the PWAS $t_{p}$ are given in Table 1. Due to the peripheral conducting and a conducted area of the quadratic PWAS, which is smaller than its outer dimensions, the effective conducted surface of the PWAS yields $A_{p}=79.875{\mathrm{mm}}^{2}$.

## 4. Experiments

**Figure 3:**
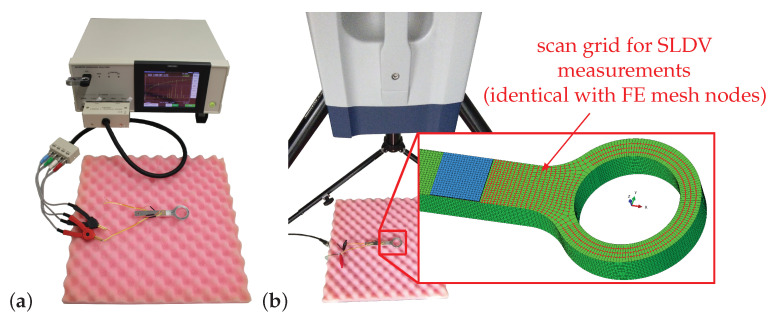
Test setups for measurements of a simplified lug (exitation at PWAS1) with (**a**) IMA (Hioki IM 3570), (**b**) SLDV (Polytec PSV-500-HV).

Two experimental setups are used to measure the frequency response of the considered simplified necked lug in the pristine and subsequently in the damaged state. In all experiments only one of the PWAS installed is excited within a frequency range of 1 kHz to 100 kHz (the second PWAS is not connected and intended for future studies). Furthermore, the sample was placed on foamed polymer typical for packing material as depicted in Figure 3.

### 4.1. Experimental Setups

The first experimental setup is depicted in Figure 3a. The excitation amplitude for the measurement with the IMA (Hioki IM 3570) is $\hat{U}=5\sqrt{2}\;V$. The frequency spectra (spectrum elements: conductance *G* and *B*) of PWAS1 are measured with a resolution of 125 Hz and for each frequency step 16 single measurement values are averaged. Figure 3b depicts the second measurement setup with the SLDV (Polytec PSV-500-HV). The excitation used for these measurements is a so-called periodic chirp signal (duration 64 ms) with an amplitude of $\hat{U}=9V$. The frequency spectra (spectrum element: out-of-plane velocity $v_{n}$) were measured with a sample rate of $f_{S}=250\mathrm{kHz}$ (yielding a frequency resolution of 15.625 Hz). The normal distance between SLDV laser head and the necked lug sample was 400 mm. The coordinates of $N_{\mathrm{SLDV}}=1005$ scan points on the upper surface (i.e., the surface in positive *z*-direction on which PWAS1 is applied, see Figure 3b) and lower surface are exported from the FE mesh. For each scan point five measurements were taken and averaged to minimize the signal-to-noise ratio.

### 4.2. Experimental Sequence

The conducted experimental investigation can be subdivided into the following three parts, which were carried out consecutively.

1. The pristine sample was measured by connecting PWAS1 to the IMA as depicted in Figure 3a. Then the pristine sample was investigated by measuring $v_{n}$ at the defined $N_{\mathrm{SLDV}}$ scan points on the upper surface using the SLDV, see Figure 3b. Subsequently, the sample was turned and the lower surface with the same amount of scan points was measured in order to better visualize the exited mode shapes.
2. After initial measurements an artificial crack of length 2 mm at $\beta_{\mathrm{in}}=90{}^{\circ }$ was introduced into the necked lug sample using a mechanical fret saw (blade thickness of 0.322 mm measured with an commercial outside micrometer).
3. Finally, the artificially damaged sample was measured in the same way as described for the pristine sample, see point 1 of this list.

## 5. Results and Discussion

**Figure 4:**
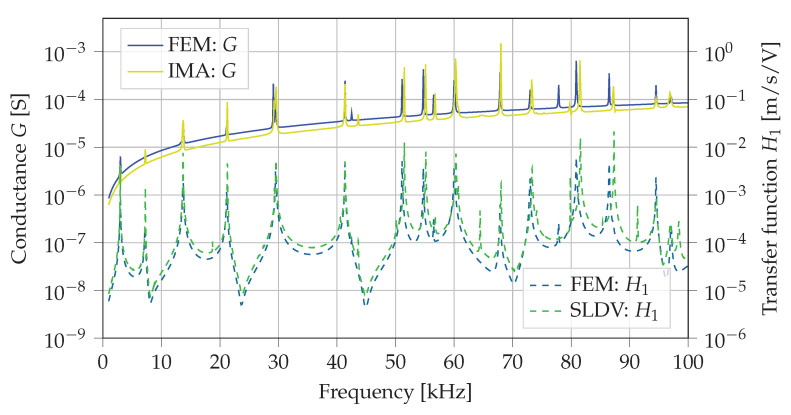
Frequency spectra of the pristine necked lug calculated with the FEM and measured with the IMA as well as with the SLDV.

The results calculated with the finite element method (FEM) and measured with the IMA as well as with the SLDV are simple frequency spectra. For comparison of calculated and measured resonance frequencies these spectra are plotted in Figure 4 on two different logarithmic scales. On the left *y*-axis the calculated and measured conductance G=G(ω) according to Equation (1) of the PWAS are compared. The right *y*-axis presents the calculated and measured mean amplitudes of the transfer function $H_{1}=H_{1}\left(\omega \right)$ defined as

(5) $$H_{1}\left(\omega \right)=\frac{1}{N_{\mathrm{SLDV}}}\sum_{n=1}^{N_{\mathrm{SLDV}}}\mathrm{abs}\left\{\frac{v_{n}\left(\omega \right)}{U(\omega )}\right\},$$

where $v_{n}\left(\omega \right)$ is the complex out-of-plane velocity of each scan point *n* of the upper surface of the lug (see detail of front section of the lug in Figure 3b). Figure 4 shows that most resonance peaks of all four spectra fit well to each other.

The calculated spectra (FEM: *G* and FEM: $H_{1}$) yield identical resonance frequencies. Between measured and calculated pristine spectra a deviation less than 1% is observed for most of the resonance frequencies. Only two resonances at 42.5 kHz and 77.875 kHz show a larger deviation of up to 2.6%. These two modes are mainly dominated by flexural in-plane bending of the ring-shaped part of the lug. The overall shapes of calculated and measured pristine spectra fit qualitatively well together. Furthermore, calculated and measured amplitudes of the pristine spectra yield values in the same order of magnitude. Therefore, the numerical model is considered to represent the real measurement setups in terms of electrical and mechanical responses in a satisfactory manner.

However, the authors emphasize that this study is based on an idealized lug (simplified geometry, no bearing in the lug hole, no residual stresses, clamping or external loading of the lug considered). While expected deviations of a true geometry to the idealized necked lug, residual stresses and the clamping of the lug’s shaft are believed to have little effect on the proposed crack identification method, the authors assume that the additional weight, stiffness and damping due to a press-fitted bearing affects the crack monitoring methodology significantly. However, the proposed method is expected to still allow an evaluation as an initiating and propagating crack would loosen the bearing, and thus, affect the dynamic response strongly due to stiffness and damping changes. This would have high impact on an applied damage metric. Furthermore, it is believed that frequency features of the lug become more prominent for an increasingly loose bearing. Therefore, this investigation represents a first step towards a crack identification for necked double shear lugs in real applications.

To address the initial questions regarding crack detection and identification possibilities with PWAS in necked lugs, a two-step analysis of the EMI measurement results is used. First, common damage metrics are used to evaluate a measured spectra with respect to a previous baseline measurement (in SHM applications typically of the undamaged state) for a simple and efficient damage detection. Second, specific spectral features of the calculated and measured frequency spectra are investigated in more detail to validate the detection result and furthermore conclude on the damage location and size. Special emphasis is given to resonance frequencies and mode shapes of the considered necked double shear lug that are most sensitive to cracks at critical locations. These are found model-based by means of coupled-field FE simulations.

### 5.1. Damage Metrics: Crack Detection

One simple way to compare different frequency spectra are damage metrics. Such damage metrics are statistical measures to identify deviations between two compared data sets. In SHM, a damage metrics yield a scalar value by directly comparing the spectra of the pristine and damaged state of a structure. Commonly used damage metrics are the root mean square deviation (RMSD), the mean absolute percentage deviation (MAPD) and the correlation coefficient deviation (CCD) which are calculated according to [7] with

(6) $$\begin{array}{cc} \; & \mathrm{RMSD}=\sqrt{\frac{\sum_{i=1}^{N_{S}}{\left(S_{i}-S_{i}^{0}\right)}^{2}}{\sum_{i=1}^{N_{S}}{\left(S_{i}^{0}\right)}^{2}}} \end{array}$$

(7) $$\begin{array}{cc} \; & \mathrm{MAPD}=\sum_{i=1}^{N_{S}}\left|\frac{S_{i}-S_{i}^{0}}{S_{i}^{0}}\right| \end{array}$$

(8) $$\begin{array}{cc} \; & \mathrm{CCD}=1-\frac{\sum_{i=1}^{N_{S}}\left(S_{i}-\bar{S}\right)\left(S_{i}^{0}-{\bar{S}}^{0}\right)}{\sqrt{\sum_{i=1}^{N_{S}}{\left(S_{i}-\bar{S}\right)}^{2}\sum_{i=1}^{N_{S}}{\left(S_{i}^{0}-{\bar{S}}^{0}\right)}^{2}}} \end{array}$$

where $N_{S}$ is the number of frequencies in the spectrum. The spectrum elements $S_{i}$ and $S_{i}^{0}$ used in this paper are either the conductance *G* for electrical responses or the transfer function $H_{1}$ for mechanical responses. The superscript 0 denotes spectral elements of the pristine structure without a crack. Mean values of spectra are indicated with $\bar{S}$ and ${\bar{S}}^{0}$.

**Figure 5:**
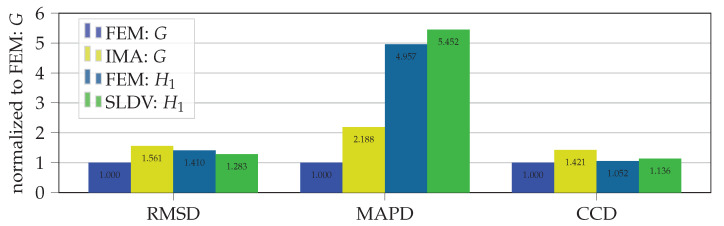
Comparison of damage metrices RMSD, MAPD and CCD for different spectrum elements (pristine vs. cracked for $a_{\mathrm{in}}=2\mathrm{mm}$, $\beta_{\mathrm{in}}=90{}^{\circ }$).

To compare pristine with damaged spectra calculated with the finite element method and measured with the impedance analyser as well as with the SLDV the damage metrics given in Equations (6)–(8) are computed for $N_{S}=793$ sample points within the frequency range of 1 kHz to 100 kHz (sample rate of 125 Hz). Figure 5 presents the damage metrics calculated with FE simulations (S=G and $S=H_{1}$) as well as IMA (S=G) and SLDV ($S=H_{1}$) measurements from the necked lug with a crack of length $a_{\mathrm{in}}=2\mathrm{mm}$ at $\beta_{\mathrm{in}}=90{}^{\circ }$ with respect to the pristine state without a crack. For comparison all calculated damage metrics are normalized by the FEM results.

The values of damage metrics RMSD and CCD yield similar results, even though spectra with different elements (*G* and $H_{1}$) are compared. However, the values of damage metric MAPD differ from each other significantly. In particular, the damage metric MAPD of calculated spectra $H_{1}$ yields a four times larger value than for the calculated conductance spectra *G*. A closer look to Equation (7) for damage metric MAPD and comparing it to the other two damage metrics could explain this deviation. The numerator of MAPD holds the spectrum element of the pristine structure for each frequency step. This is in contrast to the other two damage metrics where the numerator holds a sum of all spectrum elements, which reduces the impact of large spectral deviations at single frequencies. Therefore, further analysis and visualization of results is based on the damage metric CCD, which shows the best correlation across compared spectra (FEM: *G* and $H_{1}$, IMA: *G*, SLDV: $H_{1}$) in Figure 5.

**Figure 6:**
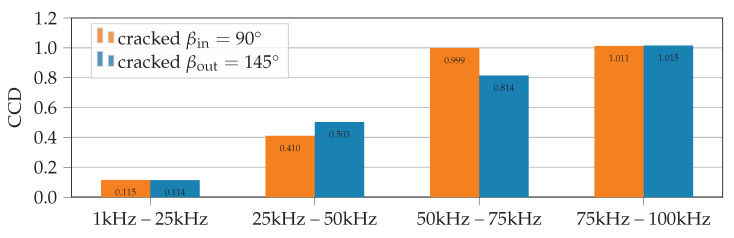
Detection of 2 mm long cracks at different crack locations with damage metric CCD. Damage metrics are calculated with spectrum element S=G from FEM results.

In Figure 6 values of damage metric CCD (computed with FEM) are compared for the two considered failure locations ($\beta_{\mathrm{in}}=90{}^{\circ }$ and $\beta_{\mathrm{out}}=145{}^{\circ }$). To identify regions of the spectra, which are most sensitive to the considered damage (crack lengths $a_{\mathrm{in}}=a_{\mathrm{out}}=2\mathrm{mm}$), the full spectrum range is divided into four parts: 1 kHz to 25 kHz, 25 kHz to 50 kHz, 50 kHz to 75 kHz and 75 kHz to 100 kHz. Subsequently, the damage metrics are calculated separately for each part of the spectrum. For both crack locations the damage metrics CCD yield highest values for frequencies above 50 kHz (this is also true for damage metrics RMSD and MAPD).

**Figure 7:**
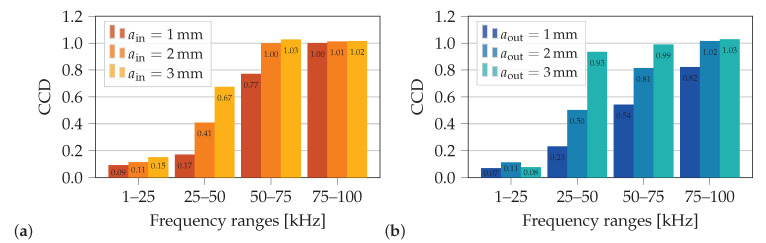
Comparison of damage metrics for growing cracks at locations (**a**) $\beta_{\mathrm{in}}=90{}^{\circ }$ and (**b**) $\beta_{\mathrm{out}}=145{}^{\circ }$. All damage metrics are calculated with spectrum element S=G from FEM results.

Similar trends are observed if the crack lengths are varied. Simulations with FE models and cracks of lengths between 1 mm to 3 mm show that the frequency ranges above 50 kHz yield the highest values for the considered damage metrics, see Figure 7.

Additionally, for most frequency ranges a strong correlation between crack length and damage metric value can be observed. Hence, to detect a crack in necked lugs the damage metrics values of frequency ranges above 50 kHz can be used. Based on these FEM results a threshold limit of CCD>0.5 in the frequency ranges above 50 kHz is proposed for detecting a crack with a minimum length of a=1mm at locations $\beta_{\mathrm{in}}=90{}^{\circ }$ and $\beta_{\mathrm{out}}=145{}^{\circ }$. This rather large threshold level is believed to be robust enough against uncertainties in real measurements (signal noise, temperature changes, etc.). In fact, damage metrics calculated by comparing the pristine spectra of independent experimental measurements with PWAS1 and PWAS2 yield values of CCD<0.1 for all frequency ranges. Nevertheless, experiments with increasing crack lengths and varying environmental influences are needed to validate the chosen crack detection threshold limit. Furthermore, the damage metric-based crack detection requires baseline measurements at the pristine structure and is prone to environmental uncertainties. The numerically calculated values of damage metrics give no clear information to evaluate if a crack initiates at the critical location on the inside or the outside surface. Therefore, spectral features of the identified highly sensitive frequency range above 50 kHz are investigated in more detail in a second analysis step.

### 5.2. Spectral Features: Crack Localization

**Figure 8:**
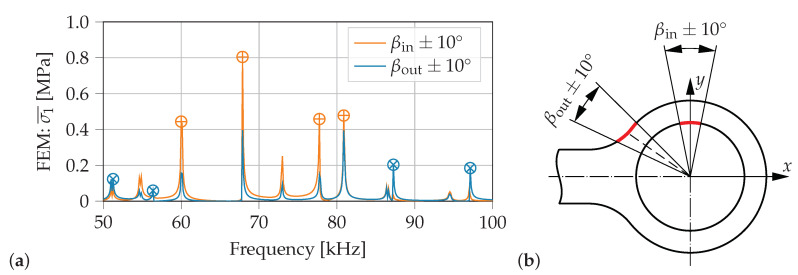
Identification of sensitive resonance frequencies: (**a**) Mean stress spectra $\bar{\sigma_{1}}$ of pristine necked lug, (**b**) FEM stress results $\sigma_{1}$ in the defined range of ±10 ${}^{\circ }$ around each location of possible crack initiation are used to calculate the mean values of major principal stresses $\bar{\sigma_{1}}$.

Besides using damage metrics, spectral features can also be used to identify structural changes [7]. In this contribution the considered structural changes are cracks, which initiate at two specific locations that show high stress concentrations at operational loads. After crack initiation cracks usually grow in directions perpendicular to the major principal stress [37]. As a crack reduces the stress in this major principal direction to zero at its face, it is assumed that vibration modes of the pristine structure that show a large major principal stress at a location of interest are also sensitive to the occurrence of a crack. Therefore, to identify resonance frequencies which are sensitive to occurring cracks at the two critical locations, spectra of mean major principal stresses $\bar{\sigma_{1}}$ are investigated. These mean stresses $\bar{\sigma_{1}}$ are calculated by averaging all FEM stress results $\sigma_{1}$, computed in coupled-field FE simulations (dynamic excitation of PWAS1 mounted on the pristine necked lug) on the surfaces over the whole thickness of the lug and within an angle of ±10 ${}^{\circ }$ around both locations of possible crack initiation ($\beta_{\mathrm{in}}=90{}^{\circ }$, $\beta_{\mathrm{out}}=145{}^{\circ }$), see Figure 8b. In Figure 8a the mean values of the major principal stresses $\bar{\sigma_{1}}$ are plotted in the frequency range of 50 kHz to 100 kHz. The spectrum of $\bar{\sigma_{1}}$ at the inside surface of possible crack initiation yields the highest resonance peaks, marked with ⊕ in Figure 8a. The most prominent resonance frequency appears at f≈68kHz. At these resonances the pristine structure is deformed according to the excited mode shapes, which results in the highest major principal stresses (averaged over the area $\beta_{\mathrm{in}}\pm 10{}^{\circ }$) compared to other exited mode shapes at other resonance frequencies. A crack initiation in the area $\beta_{\mathrm{in}}\pm 10{}^{\circ }$ significantly reduces the local stiffness of the structure in this region. Hence, for the resonance frequencies marked with ⊕ in Figure 8 it is assumed that they are sensitive to cracks initiating at $\beta_{\mathrm{in}}\pm 10{}^{\circ }$. Similar can be said for the spectrum of $\bar{\sigma_{1}}$ at the location of possible crack initiation at the outside surface $\beta_{\mathrm{out}}\pm 10{}^{\circ }$. This spectrum yields smaller amplitudes for most frequencies. However, four resonance frequencies at f≈51kHz, 56 kHz, 87 kHz and 97 kHz have higher stress amplitudes $\bar{\sigma_{1}}$ at the outside surface of possible crack initiation than at the inside surface of possible crack initiation (marked with ⊗ in Figure 8a).

Hence, it is assumed that a monitoring of at least two resonance frequencies with contrasting sensitivities to cracks at $\beta_{\mathrm{in}}\pm 10{}^{\circ }$ and $\beta_{\mathrm{out}}\pm 10{}^{\circ }$ can be used to distinguish between a crack initiation from the inside or outside surface. The four resonance frequencies each, identified in Figure 8 to highlight both locations of possible crack initiation, are listed in the first two columns of Table 2.

**Figure 9:**
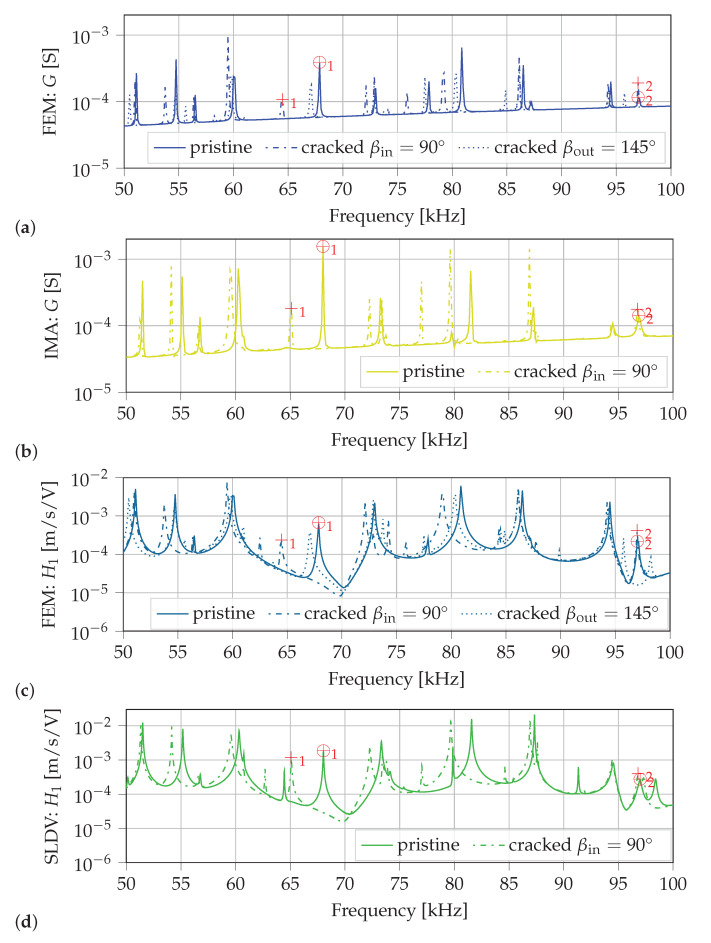
Spectra of pristine and damaged (cracks of length 2 mm) necked lug of (**a**) conductance simulated with FEM; (**b**) conductance measured with the IMA; (**c**) mean out-of-plane vibration results simulated with FEM; (**d**) mean out-of-plane vibration results measured with the SLDV. Resonance frequency shifts $\Delta f_{1}$ and $\Delta f_{2}$ to identify the distinct crack location are highlighted with ⊕_1,2_ in pristine spectra and with +_1,2_ in spectra of the cracked lug ($a_{\mathrm{in}}=2\mathrm{mm}$, $\beta_{\mathrm{in}}=90{}^{\circ }$).

Subsequently, in Figure 9 pristine and damaged (cracks of length 2 mm at locations $\beta_{\mathrm{in}}=90{}^{\circ }$ and $\beta_{\mathrm{out}}=145{}^{\circ }$) state spectra are plotted in the frequency range of 50 kHz to 100 kHz. Figure 9a,c show frequency spectra calculated with coupled-field FE simulations. Figure 9b,d show frequency spectra measured with the IMA and the SLDV, respectively.

As expected, at frequencies with the highest stress amplitudes $\bar{\sigma_{1}}$ (marked with ⊕ in Figure 8a) the resulting shifts of resonance frequencies between pristine and cracked state are larger for the inside crack than for the outside crack, see also Table 2. In contrast, the identified frequencies where the stress amplitudes $\bar{\sigma_{1}}$ for the outside crack are larger than for the inside crack (marked with ⊗ in Figure 8a) the frequency shifts for the outside crack are significantly larger. Moreover, these resonance frequencies are almost insensitive for a crack at $\beta_{\mathrm{in}}=90{}^{\circ }$, which is clearly illustrated in Table 2. This special behavior can be used to differentiate between the two critical crack locations of the considered structure. Frequency shifts $\Delta f=f_{\mathrm{cracked}}-f_{\mathrm{pristine}}$ for resonance frequencies marked with ⊕ (largest major principal stress around $\beta_{\mathrm{in}}=90{}^{\circ }$), which are larger than frequency shifts at resonance frequencies marked with ⊗ (largest major principal stress around $\beta_{\mathrm{out}}=145{}^{\circ }$), indicate a crack growth starting from the inside surface. In contrast, for a crack initiating at $\beta_{\mathrm{out}}=145{}^{\circ }$ the frequency shifts at resonance frequencies marked with ⊗ are larger or at least in a similar range compared to frequency shifts at resonance frequencies marked with ⊕. Therefore, a monitoring of only two resonance frequencies with contrasting sensitivities for the two crack locations is sufficient to identify the distinct crack location for the two considered possibilities. However, employing more sensitive resonance frequencies can increase the reliability of the localization or allow the differentiation between more damage locations.

**Figure 10:**
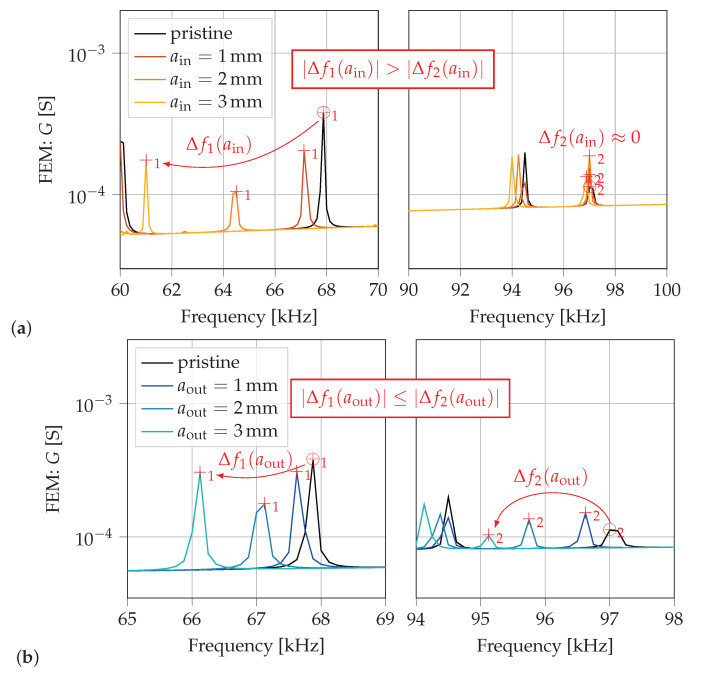
Comparison of numerically calculated conductance spectra for growing cracks at location (**a**) $\beta_{\mathrm{in}}=90{}^{\circ }$ and (**b**) $\beta_{\mathrm{out}}=145{}^{\circ }$. Detailed plot of frequency spectra around each resonance frequency. Relations between frequency shifts $\Delta f_{1}$ and $\Delta f_{2}$ is based on absolute values.

To illustrate the proposed crack localization method two resonance frequencies with contrasting sensitivities are chosen from Table 2. The influence of growing cracks at each location on simulated FEM conductance spectra (separate simulations for each crack length and location) is analyzed around these two resonance frequencies: $f_{1,\mathrm{pristine}}=67.875\mathrm{kHz}$ is highly sensitive to a crack at $\beta_{\mathrm{in}}=90{}^{\circ }$ and less sensitive to a crack at $\beta_{\mathrm{out}}=145{}^{\circ }$; $f_{2,\mathrm{pristine}}=97\mathrm{kHz}$ is highly sensitive to a crack at $\beta_{\mathrm{out}}=145{}^{\circ }$ and almost insensitive to a crack at $\beta_{\mathrm{in}}=90{}^{\circ }$. In Figure 9 and Figure 10 the corresponding resonance frequency peaks are highlighted with ⊕_1,2_ and +_1,2_ of the pristine and cracked states, respectively.

The spectra of a growing crack at $\beta_{\mathrm{in}}=90{}^{\circ }$ are depicted in Figure 10a (one graph for each chosen resonance frequency with adapted *x*-axis scaling for better visualization). At this crack location the first chosen resonance frequency $f_{1}$ shows a significant shift of resonance peaks $\Delta f_{1}\left(a_{\mathrm{in}}\right)$ to lower frequencies and the second resonance frequency $f_{2}$ shows almost no shift in frequencies ($\Delta f_{2}\left(a_{\mathrm{in}}\right)\approx 0$) due to a growing crack (considering absolute values). In contrast, for a developing crack at $\beta_{\mathrm{out}}=145{}^{\circ }$, both chosen resonance frequencies $f_{1}$ and $f_{2}$ show similar frequency shifts (in absolute values) to lower frequencies, see Figure 10b. Nevertheless, for all crack lengths $a_{\mathrm{out}}$ the absolute values of frequency shifts satisfy the condition $|\Delta f_{1}\left(a_{\mathrm{out}}\right)\left|\leq \right|\Delta f_{2}\left(a_{\mathrm{out}}\right)|$. Hence, if $|\Delta f_{1}\left|>\right|\Delta f_{2}|$ a crack at $\beta_{\mathrm{in}}=90{}^{\circ }$ is indicated and if $|\Delta f_{1}\left|\leq \right|\Delta f_{2}|$ a crack at $\beta_{\mathrm{out}}=145{}^{\circ }$ is indicated.

The performed experiments show similar results and validate the methodology to identify the crack localization using two resonance frequency shifts, see Table 3. In Figure 9 the overall shapes of calculated and measured spectra of the cracked necked lug ($a_{\mathrm{in}}=2\mathrm{mm}$, $\beta_{\mathrm{in}}=90{}^{\circ }$), qualitatively compared, fit well together. Resonance frequencies of measured and calculated spectra of the cracked lug show a similar (small) deviation as observed for the spectra of the pristine lug. To experimentally demonstrate the crack localization both chosen resonance frequency shifts in calculated and measured spectra are presented in Table 3. It is clearly seen, that in measured and calculated spectra $|\Delta f_{1}|>4$%, whereas $|\Delta f_{2}|\approx 0$%, which indicates a crack at $\beta_{\mathrm{in}}=90{}^{\circ }$.

The most significant shifts of resonance frequencies in calculated and measured spectra happens for the resonance frequency f≈68kHz of the pristine necked lug, see Figure 9 and Figure 10. This resonance frequency is highly sensitive to a crack at $\beta_{\mathrm{in}}=90{}^{\circ }$ and also shows a significant frequency shift for a crack at $\beta_{\mathrm{out}}=145{}^{\circ }$, see Table 2. Therefore, in order to estimate the crack size this specific resonance frequency is analyzed in more detail.

### 5.3. Spectral Features: Crack Size

**Figure 11:**
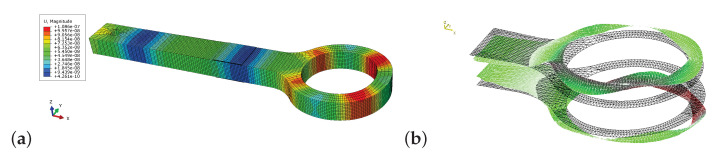
Comparison of in-plane radial vibration mode shape of pristine necked lug at (**a**) $f_{\mathrm{pristine}}^{\mathrm{FEM}}$ = 67,875 Hz for FEM and (**b**) $f_{\mathrm{pristine}}^{\mathrm{SLDV}}$ = 68,031 Hz for SLDV.

The mode shape for the resonance frequency f≈68kHz of the pristine necked lug is depicted in Figure 11.

In the FEM contour plot it can be seen that the shaft of the necked lug vibrates axially and the ring-shaped part of the lug vibrates in-plane radially with four nodes, see Figure 11a. With the SLDV only out-of-plane deformations can be measured, and hence in Figure 11b the transverse thickness deformation due to the in-plane radial vibration is visualized. This specific resonance frequency, where the ring-shaped part of the lug vibrates predominately in-plane with radial displacements shall be studied analytically. For this purpose the necked lug depicted in Figure 1 is roughly split up into a straight bar and a simple ring. To approximate the relation between the vibration frequency and the geometry of the ring we simply consider an in-plane purely radial vibration. With a mean radius of $r_{m}=\left(D+d\right)/4$ the natural frequency is [38]

(9) $$f_{\mathrm{radial}}=\frac{1}{2\pi r_{m}}\sqrt{\frac{E}{\rho }}.$$

Given the dimensions of the ring-shaped front end of the reference lug, as described in Section 2, the natural frequency of the in-plane radial vibration of a simple ring yields $f_{\mathrm{radial}}=64.782\mathrm{kHz}$. This analytically calculated natural frequency is actually close to the resonance frequency of the lug at 67.875 kHz even though the shaft of the lug is neglected by Equation (9). Also for necked lugs with similar geometry but different mean ring radius $r_{m}$ such an in-plane mode shape exists, which has a resonance frequency close to the in-plane radial resonance frequency of a corresponding simple ring. This specific resonance frequency changes reciprocally proportional with $r_{m}$, as shown in Table 4, which is also characterized by Equation 9. Additionally, the analytic in-plane radial resonance frequency of the simple ring is independent of the ring thickness, see Equation (9). Subsequently, changing the thickness *t* of the lug has small influence on the numerically calculated radial resonance frequencies of the necked lug, see Table 4. However, for lugs with different thickness or mean radius this specific in-plane radial mode shape is highly sensitive to radial cracks.

**Figure 12:**
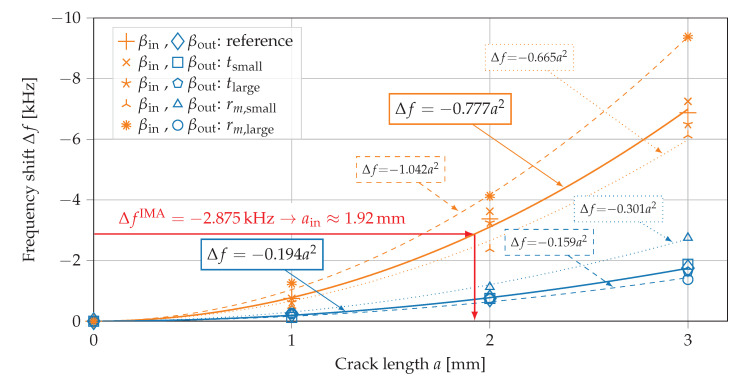
Shift of resonance peaks Δf due to growing cracks at $\beta_{\mathrm{in}}=90{}^{\circ }$ and $\beta_{\mathrm{out}}=145{}^{\circ }$ in necked lugs with different geometries according to Table 4.

As already presented for the reference lug geometry, a growing crack at $\beta_{\mathrm{in}}=90{}^{\circ }$ shows a much larger deviation of resonance peaks than a crack at $\beta_{\mathrm{out}}=145{}^{\circ }$, see Figure 10. For different geometries, this behavior can be displayed well if the frequency shifts $\Delta f=f_{\mathrm{cracked}}-f_{\mathrm{pristine}}$ of considered resonance frequencies are plotted over the crack length, see Figure 12.

For both crack locations a nonlinear relationship between crack length and resulting frequency shift are found. For the reference lug geometry (t=6mm, $r_{m}=12.41\mathrm{mm}$, a= 1 mm to 3 mm) a least squares fit to quadratic functions yields resonance frequency shifts of $\Delta f=\lambda a^{2}$ with $\lambda =-0.777\mathrm{kHz}/{\mathrm{mm}}^{2}$ for the inside crack at $\beta_{\mathrm{in}}=90{}^{\circ }$ and $\lambda =-0.194\mathrm{kHz}/{\mathrm{mm}}^{2}$ for the outside crack at $\beta_{\mathrm{out}}=145{}^{\circ }$. Similar trends are shown for other geometries with varied $r_{m}$ in Figure 12. For a larger mean radius the sensitivity of frequency shifts is increased for growing cracks on the inside surface at $\beta_{\mathrm{in}}=90{}^{\circ }$ (c.f. $\lambda =-1.042\mathrm{kHz}/{\mathrm{mm}}^{2}$ for $r_{m}=13.41\mathrm{mm}$) and decreased for growing cracks on the outside surface at $\beta_{\mathrm{out}}=145{}^{\circ }$ (c.f. $\lambda =-0.159\mathrm{kHz}/{\mathrm{mm}}^{2}$ for $r_{m}=13.41\mathrm{mm}$). In contrast, the sensitivities change in the opposite direction for a smaller mean radius. In this case the sensitivity is smaller for a crack growth on the inside surface at $\beta_{\mathrm{in}}=90{}^{\circ }$ (c.f. $\lambda =-0.665\mathrm{kHz}/{\mathrm{mm}}^{2}$ for $r_{m}=11.41\mathrm{mm}$) and larger for crack growth on the outside surface at $\beta_{\mathrm{out}}=145{}^{\circ }$ (c.f. $\lambda =-0.301\mathrm{kHz}/{\mathrm{mm}}^{2}$ for $r_{m}=11.41\mathrm{mm}$). However, a change in the thickness *t* of the lug has small influence on the frequency shifts due to crack growth, see Figure 12. We conclude that an estimation of crack length with the trends of frequency shifts (of mode shape at f≈68kHz) due to crack growth is robust against manufacturing uncertainties of the thickness and also can be used for similar necked lug geometries with varying mean radius $r_{m}$. For other mode shapes such clear trends of frequency shifts related to crack growth were not observed. The quadratic trend of frequency shifts due to crack growth were only found for the in-plane radial mode shape presented in Figure 11. The authors assume that the quadratic trends of frequency shifts are directly related to the reduced stiffness of the ring-shaped part of the lug and that the location of the stiffness reduction (inside at $\beta_{\mathrm{in}}=90{}^{\circ }$ or outside at $\beta_{\mathrm{out}}=145{}^{\circ }$) defines the strongly different effect on the considered mode shape and resonance frequency.

The presented model-based method (developed using coupled field FE simulations) is now checked using experimental measurements. Given the frequency shift of $\Delta f^{\mathrm{IMA}}=-2.875\mathrm{kHz}$ measured with the impedance analyzer yields an estimated crack size of $a_{\mathrm{in}}=1.92\mathrm{mm}$, see Figure 12. This result fits well to the introduced crack length of 2 mm. The small deviation in estimated crack length may result from the finite width of the introduced crack (compared to zero crack width in FE models) or manufacturing deviations (e.g., actual crack length, roundness of lug ring).

## 6. Conclusions

A variety of FE simulations as well as measurements with an IMA and a SLDV were performed to investigate the EMI-based crack detection and identification in necked double shear lugs. The developed FE models for coupled-field simulations yield frequency spectra which fit well to all experimental measurements in the considered frequency range of 1 kHz to 100 kHz. The calculated and measured resonance frequencies of conductance *G* and transfer function $H_{1}$ show less than 1% deviation, except for two in-plane flexural modes at 42.5 kHz and 77.875 kHz which show a deviation up to 2.6%.

Analyzed frequency spectra from FE simulations and measurements clearly reflect artificially introduced through-cracks in the considered necked lug. The two critical locations of crack initiation (inside at the bolt hole at $\beta_{\mathrm{in}}=90{}^{\circ }$ and outside at the transition region between shaft and largest diameter of the lug at $\beta_{\mathrm{out}}=145{}^{\circ }$) have been investigated.

In a first analysis step, common damage metrics were investigated to identify regions of the spectrum, which are most sensitive to through-cracks at both locations. Thus, the full frequency range was separated in four parts (1 kHz to 25 kHz, 25 kHz to 50 kHz, 50 kHz to 75 kHz and 75 kHz to 100 kHz) and damage metrics were evaluated for each individual range. All calculated damage metrics (CCD, RMSD and MAPD) for both crack locations yield the highest values for frequencies above 50 kHz and reflect the presence of a crack. The CCD metric shows best comparability between numerical and experimental results. In the considered frequency ranges above 50 kHz a crack length of 1 mm could be readily detected if the damage metric CCD exceeds a proposed threshold level of CCD>0.5. This comparatively high threshold level is assumed to be robust against environmental influences (e.g., measurement noise, temperature changes). Hence, crack detection in necked lugs is possible by simply evaluating the damage metric CCD in frequency ranges of 50 kHz to 75 kHz and 75 kHz to 100 kHz.

In a second step, the spectral features are analyzed within the identified frequency range, which is highly sensitive to crack initiation. Initially, spectra of the mean values of major principal stresses around both considered crack locations are investigated by the FE model. A comparison of the amplitude of each resonance peak indicates if the corresponding resonance frequency is sensitive to a crack at the inside or outside surface. This model-based approach yields two frequencies, which show different sensitivities to the considered critical crack locations. Together with monitoring their frequency shifts Δf the distinct crack location can be identified for both numerical and experimental results. Additionally, spectral features that show one of the two expected changes of the resonance frequency verify the damage-metric-based crack detection of first evaluation step.

Subsequently, the resonance peak of the necked lug, which actually yields a frequency close to the analytically calculated in-plane radial vibration frequency of a simple ring (with corresponding dimensions), is found to be highly sensitive to crack formation at both critical locations. This effect is further analyzed to estimate the crack size. The specific resonance shows the highest frequency shifts due to growing cracks at $\beta_{\mathrm{in}}=90{}^{\circ }$ and is also strongly affected by cracks at $\beta_{\mathrm{out}}=145{}^{\circ }$. Monitoring this specific resonance frequency yields clear relationships between frequency shift Δf and length *a* of a crack at both considered critical locations, allowing a model-based crack size estimation for the experimental measurements. The identification of crack location and size is done in a model-based approach using an adequate FE model, and hence is expected to be suitable for other geometries similar to necked double shear lugs.

Consequently, the EMI method shows high potential for structural health monitoring of necked double shear lugs, and other structural components with ring-shaped topology and similar damage initiation and propagation, on different levels. Besides pure detection of cracks it is also possible to distinguish between their locations. Furthermore, with the presented method a crack’s length can be quantified. This allows estimating the corresponding stress intensity factors for operational loads, which finally enables the prognosis of the remaining fatigue life of necked double shear lugs.

However, in its current state, the method requires baseline measurements at all levels. Observing changes in the frequency features might also allow a model-based damage detection in the future. Moreover, further research has to be done to advance the results of this first study for use in real applications. The crack growth process has to be investigated for other typical crack shapes (quarter circular and elliptical corner cracks) and naturally initiated cracks during fatigue loading. Furthermore, bushings or spherical bearings in the lug hole as well as loading during measurements and changing temperatures will influence the frequency response spectra. To reach sufficient reliability for real applications, the presented crack detection approach for necked double shear lugs using PWAS and the EMI method have to be validated under such conditions.

## Figures and Tables

**Table 1:** Properties of PWAS (material: PIC151) according to supplier [34].

Physical and dielectrial properties			
	Edge length $w_{p}$	10	mm
	Thickness $t_{p}$	0.25	mm
	Density $\rho_{p}$	7800	$\mathrm{kg}/m^{3}$
	Poisson’s ratio $\nu_{p}$	0.34	-
	Elastic compliance coefficient in-plane $s_{11}^{E}$	15 × 10^−12^	$m^{2}/N$
	Elastic compliance coefficient out-of-plane $s_{33}^{E}$	19 × 10^−12^	$10^{-12}$ $m^{2}/N$
	Relative permittivity in the polarization $\varepsilon_{33}^{T}$	2400$\;\varepsilon_{0}$	As/Vm
	Relative permittivity in direction perpendicular to polarity $\varepsilon_{11}^{T}$	1980$\;\varepsilon_{0}$	As/Vm
	Dielectric loss factor tanδ	20 × 10^−3^	-
**Electro-mechanical properties**			
	Piezoelectric charge coefficient $d_{31}$	−210 × 10^−12^	C/N
	Piezoelectric charge coefficient $d_{33}$	500 × 10^−12^	C/N

**Table 2:** Comparison of resonance frequencies in the conductance spectra *G* calculated with FEM for both crack locations. The relative shifts of resonance frequencies for each crack location are highlighted in orange and blue, respectively.

		$a_{\mathrm{in}}=2\;\mathrm{mm},$$\beta_{\mathrm{in}}$ = 90°			$a_{\mathrm{out}}=2\;\mathrm{mm},$$\beta_{\mathrm{in}}$ = 145°		
Marker	$f_{\mathrm{pristine}}$	$f_{\mathrm{cracked}}$	Δf		$f_{\mathrm{cracked}}$	Δf	
in Figure 8	[Hz]	[Hz]	[Hz]	[%]	[Hz]	[Hz]	[%]
⊗	51,125	51,000	−125	−0.24	50,500	−625	−1.22
⊗	56,500	56,375	−125	−0.22	55,625	−875	−1.55
⊕	60,000	59,500	−500	−0.83	59,750	−250	−0.42
⊕	67,875	64,500	−3375	−4.97	67,125	−750	−1.10
⊕	77,875	75,875	−2000	−2.57	77,500	−375	−0.48
⊕	80,875	79,250	−1625	−2.01	80,375	−500	−0.62
⊗	87,250	87,125	−125	−0.14	84,875	−2375	−2.72
⊗	97,000	97,000	0	0.00	95,750	−1250	−1.29

**Table 3:** Chosen resonances $f_{1}$ and $f_{2}$ of pristine and cracked state ($a_{\mathrm{in}}=2\mathrm{mm}$, $\beta_{\mathrm{in}}=90{}^{\circ }$) from simulation and experiments to identify the crack location.

	$f_{1,\mathrm{pristine}}$	$f_{1,\mathrm{cracked}}$	$\Delta f_{1}$		$f_{2,\mathrm{pristine}}$	$f_{2,\mathrm{cracked}}$	$\Delta f_{2}$	
	[Hz]	[Hz]	[Hz]	[%]	[Hz]	[Hz]	[Hz]	[%]
FEM: *G*, $H_{1}$	67,875	64,500	−3375	−4.97	97,000	97,000	0	0.00
IMA: *G*	68,000	65,125	−2875	−4.23	96,875	96,750	−125	−0.18
SLDV: $H_{1}$	68,031	65,094	−2937	−4.32	96,984	96,797	−187	−0.28

**Table 4:** Comparison of resonances frequencies of in-plane radial vibration computed analytically with Equation (9) and FEM simulation for different lug geometries.

	$r_{m}$	*t*	$f_{\mathrm{pristine}}^{\mathrm{analytic}}$	$f_{\mathrm{pristine}}^{\mathrm{FEM}}$
Geometry	[mm]	[mm]	[Hz]	[Hz]
reference	12.41	6	64,782	67,875
$t_{\mathrm{small}}$	12.41	3	64,782	68,000
$t_{\mathrm{large}}$	12.41	9	64,782	67,750
$r_{m,\mathrm{small}}$	11.41	6	70,460	70,750
$r_{m,\mathrm{large}}$	13.41	6	59,951	64,625

## Data Availability

The data presented in this study are openly available in Zenodo at doi:10.5281/zenodo.4388169.

## Abbreviations

The following abbreviations are used in this manuscript:

SHM	Structural Health Monitoring
NDT	Non-Destructive Testing
EMI	Electro-Mechanical Impedance
PWAS	Piezoelectric Active Wafer Sensor
RMSD	Root Mean Square Deviation
MAPD	Mean Absolute Percentage Deviation
CCD	Correlation Coefficient Deviation
FE	Finite Element
FEM	Finite Element Method
IMA	Impedance Analyzer
SLDV	Scanning Laser Doppler Vibrometer
